# Severe Scrub Typhus with Acute Kidney Injury: Urine PCR Evidence from an East Coast Malaysian Cluster

**DOI:** 10.3390/tropicalmed10080208

**Published:** 2025-07-25

**Authors:** Siti Roszilawati Ramli, Nuridayu Arifin, Mohd Fahmi Ismail, Shirley Yi Fen Hii, Nur Suffia Sulaiman, Ernieenor Faraliana Che Lah, Nik Abdul Hadi Nik Abdul Aziz

**Affiliations:** 1 Bacteriology Unit, Infectious Diseases Research Centre, Institute for Medical Research, National Institutes of Health, Shah Alam 40170, Selangor, Malaysia; 2 Pekan District Health Office, Ministry of Health, Pekan 26600, Pahang, Malaysia; 3 School of Medicine and Health Sciences, Monash University Malaysia, Bandar Sunway 47500, Selangor, Malaysia; 4 Acarology Unit, Infectious Disease Research Centre, Institute for Medical Research, National Institutes of Health, Shah Alam 40170, Selangor, Malaysia; 5 Internal Medicine Department, Hospital Tengku Ampuan Afzan, Jalan Tanah Putih, Kuantan 25100, Pahang, Malaysia

**Keywords:** severe scrub typhus, cluster case, Malaysia, *Orientia tsutsugamushi*

## Abstract

**Background:** Scrub typhus (ST) is caused by *Orientia tsutsugamushi* (OT) infection, which is transmitted to humans through the bites of infected chiggers. The clinical presentations range from mild to life-threatening multi-organ dysfunction. This report describes a cluster of ST cases involving five oil palm estate workers in Pekan district, Pahang, Malaysia. **Methods:** The clinical history, laboratory, and entomological investigation were conducted on the patients, including the index case and four suspected cases in the cluster. Polymerase chain reaction (PCR) tests for OT and genotyping were performed on the patients’ blood and urine samples. Serological testing by indirect immunoperoxidase (IIP) test against Rickettsial diseases was also conducted. **Principal Findings:** Patients presented with fever, myalgia, headache, rash, cough, and eschar. The index case developed severe ST complicated by acute kidney injury (AKI) and respiratory distress, requiring intubation and ventilation at the intensive care unit of a tertiary hospital. ST was confirmed through PCR analysis of a urine sample, showcasing a novel diagnostic approach. The other four cases were confirmed by a four-fold rise in immunoglobulin G (IgG) antibody titers. **Conclusions:** Oil palm estate workers are at high risk for chigger exposure in Malaysia. Awareness among clinicians and the public of ST is crucial for effective prevention, accurate diagnosis, and optimal management.

## 1. Author Summary

Scrub typhus (ST) is a disease caused by bacteria called *Orientia tsutsugamushi* (OT), which is spread to humans through bites from infected chiggers (tiny mite larvae). While some people may experience mild symptoms, others can develop severe complications affecting multiple organs. In this report, we describe a cluster of ST cases among five oil palm estate workers in Pahang, Malaysia. The patients experienced symptoms such as fever, muscle pain, headache, rash, cough, and a distinctive scab-like wound called an eschar. One worker developed a severe form of the disease, leading to kidney failure and breathing difficulties, requiring intensive care. Diagnosis was confirmed using a specialized genetic test, polymerase chain reaction (PCR), on urine samples. The other cases were confirmed through blood tests measuring immune responses. Our findings highlight that oil palm estate workers in Malaysia are at high risk of ST due to their exposure to infected chiggers. Raising awareness among healthcare professionals and the public is essential for early diagnosis, proper treatment, and effective prevention strategies.

## 2. Introduction

Scrub typhus, or tsutsugamushi disease, is a vector-borne infection caused by OT and transmitted to humans through the bite of infected chigger mites. Previously, OT was the only recognized species within the genus; however, two more species have been discovered to cause ST, which are *Orientia chuto* and *Candidatus Orientia chiloensis* [1,2].

Malaysia’s earliest reported ST case in 1915 was linked to trombiculid mites. Over time, accumulating evidence and case reports have confirmed the disease’s endemicity in both East and West Malaysia. Until now, ST remains a persistent threat, despite the availability of modern antibiotics. There has been an apparent resurgence of cases in Malaysia, raising concerns about its increasing prevalence. There are many factors that may contribute to the upward trend of cases, which include climate change, alteration of the habitat, and distribution of the chigger mite population due to increased deforestation and construction works. The expansion of agricultural activities may increase the exposure of humans to the chiggers’ natural habitat and potentially heighten the risk of transmission. Previous studies in Korea showed that deforestation activities caused environmental changes, resulting in a significant association with an increase in the incidence of scrub typhus [3]. Kwa et al. reported that environmental changes stemming from altered land use patterns for agricultural activities such as large-scale cultivation of rice, rubber, and oil palm have contributed to an increase in vector-borne diseases, including dengue, filariasis, and scrub typhus in Malaysia. In particular, the establishment of extensive oil palm plantations has significantly impacted the ecology of chiggers and rodent populations, leading to a higher incidence of scrub typhus among high-risk groups, notably plantation workers and nearby residents [4]. On the other hand, the advancements in diagnostic technologies, including molecular and rapid test methods, have improved the detection of ST, leading to more accurate case identification. The interplay of these factors underscores the need for continued surveillance, public health interventions, and increased awareness among healthcare professionals to reduce ST cases in Malaysia.

## 3. Cluster of Cases

A cluster of ST cases was identified in a oil palm estate in the district of Pekan in Pahang, an east-coast state in Peninsular Malaysia, on 7 December 2023. The Pekan District Health Office was alerted to two suspected leptospirosis cases among oil palm workers. The index case was a 22-year-old immigrant worker of Indian nationality who worked as a fruit picker and cleaner at the oil palm estate in Pekan. Until 31 January 2024, a total of five people had a similar illness among the oil palm estate workers, which was later diagnosed as ST cases. The oil palm estate was divided into three zones, i.e., Zone 1 covered 319 hectares, while Zones 2 and 3 covered 170 and 70 hectares, respectively. All patients worked in Zone 2. The oil palm estate experienced a surge in rodent infestation from a neighboring estate before the incident. The estate had 26 workers, consisting of four supervisors, nine immigrant workers, and 13 aboriginal workers. All workers lived in houses built in the oil palm estate, except for six of the aboriginal workers who lived outside of the estate. The nearest health facilities were the Tanjung Pulai Rural Clinic, 3.4 km away, and the Padang Rumbia Health Clinic, which was 27 km away. The Pekan Hospital in Pekan town was 38 km away, while the Hospital Tengku Ampuan Afzan in Kuantan city was 58 km away from the estate.

## 4. Index Case

A 22-year-old male oil palm estate immigrant worker of Indian nationality presented on 4 December 2023 at a private clinic in the Pekan district with a complaint of fever, headache, and cough for a day. He was diagnosed with viral fever and given medication. Still unwell, he was sent by his supervisor to the Bandar Pekan Health Clinic on 7 December 2023 with the same complaint. He was tested for a full blood count, which showed findings within the normal range. He was diagnosed again with viral fever and sent home with medication.

On 9 December 2023, his conditions worsened, and he had an episode of upward rolling of the eyes with no jerking movements. He was sent to Hospital Tengku Ampuan Afzan (HTAA) in Kuantan city for further management. There, he complained of fever for a week with chills, rigor, headache, and generalized myalgia at home, even with paracetamol intake. He also had a productive cough with yellowish sputum for a week with shortness of breath for three days. This was associated with reduced effort tolerance and poor oral intake. History was taken from his friend due to a language barrier. His temperature was 39.2 °C, and there were no palpable cervical lymph nodes on admission. A chest X-ray showed bibasal haziness suggestive of pleural effusions. He was admitted into the medical ward on 10 December 2023 with a diagnosis of severe leptospirosis with pulmonary involvement and treated with intravenous (IV) ceftriaxone. The next day, the leptospirosis immunoglobulin (IgM) rapid serology test was found to be positive, and his antibiotic was changed to IV penicillin C.

On 12 December 2023, due to a desaturation of oxygen levels, he was intubated, sedated, and moved to the intensive care unit (ICU). IV methylprednisolone started and given for three days, and penicillin was stopped. A blood film for malaria parasite (BFMP) test was undertaken, which was negative. On 14 December 2023 (5th day of admission), he was febrile but had no thrombophlebitis. He was diagnosed with hospital-acquired pneumonia (HAP) and treated with IV tazocin. A viral screening and a septic workup were carried out, which included a PCR urine test for leptospirosis and ST. The patient was still febrile with a temperature of 38 °C on 16 December 2023 (7th day of admission). Culture and sensitivity tests of tracheal aspirate showed growth of multidrug-resistant *Acinetobacter baumanni,* and the patient was treated with IV colistin (IV tazocin was stopped). On 20 December 2023, the patient was still ventilated but not sedated, and IV ceftriaxone was restarted. An ultrasound of the abdomen showed no intra-abdominal fluid collection. On 21 December 2023, the patient was diagnosed with ventilation-associated pneumonia (VAP), secondary to *Pseudomonas aeruginosa* isolated from tracheal aspirates, and IV cefepime was started, as IV colistin was completed for five days. On 22 December 2023, the patient was extubated, antibiotics were stopped, and he was transferred to a general medical ward. On 27 December 2023, thrombophlebitis was seen on his left hand, and IV Unasyn treatment was started. On 28 December 2023, his temperature finally settled. The patient was on nasogastric tube feeding.

**Figure 1:**
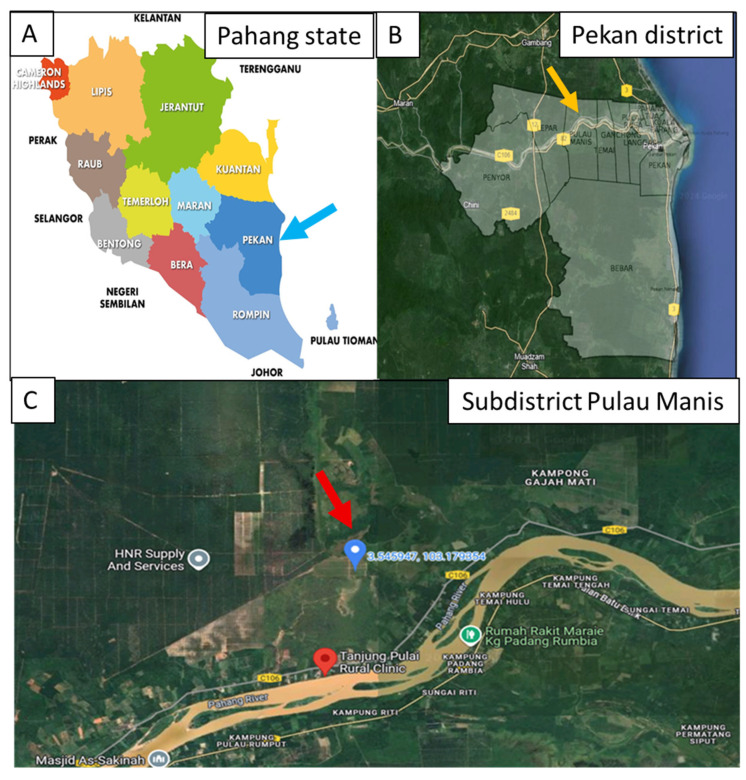
(**A**) Map of districts in Pahang; Pekan is marked with blue arrow. (**B**) The cluster involved Pekan District and subdistrict Pulau Manis (yellow arrow). (**C**) The cluster happened in Ladang Felcra RTK Tanjung Pulai, 2.5 km northeast from Tanjung Pulai Rural Clinic (marked with red arrow).

He was later discharged on 30 December 2023 to his residence, which was a shared housing facility at a oil palm estate in the Pekan district. Urine PCR test for OT was found to be positive. The indirect immunoperoxidase (IIP) test for rickettsial disease performed on 27 December 2023 and on 16 January 2024 showed a four-fold rise of immunoglobulin G against OT titers, which confirmed serological evidence of a recent ST infection. His final diagnosis was severe ST complicated by acute kidney injury (AKI) and VAP. The maps of Pahang state, Pekan districts and Pulau Manis subdistrict were illustrated in Figure 1.

## 5. Case Verification

A rapid assessment team (RAT) was sent to the location for the purpose of verification of cases. All reported cases were investigated by a dedicated team from the Pekan District Health Office. History was taken of patients’ movements within 21 days before manifestation of signs and symptoms. A suspected case was defined as any individual working at RTK Tanjung Pulai oil palm estate, Pekan, who experienced a sudden onset of fever associated with any symptoms such as headache, myalgia, rash, joint pain, abdominal pain, chigger bite scar (eschar), or reduced appetite between 3rd December 2023 and 6 February 2024. A confirmed case was defined as a suspected case with laboratory confirmation by either positive OT PCR or confirmation of serology test for Rickettsial disease [5].

All RTK Tanjung Pulai oil palm estate staff were screened to detect cases (active case detection). Case detection activities were carried out among all estate workers and spouses who resided at the oil palm estate. There were six other workers who fulfilled the criteria for a suspected case. One of the workers had similar symptoms in early November 2023. Blood samples were taken on 27 December 2023 from five workers who fulfilled the criteria of a suspected case. Samples were sent to the Bacteriology Unit, Institute for Medical Research (IMR).

All positive cases were given antibiotic treatment. The shared housing facility and its surroundings were sprayed with K-Othrine insecticide to eliminate any carrier vector. Entomological control in the estate area was carried out until 25 February 2024 (after floodwaters receded at the road to the estate). All health clinics in Pekan were alerted to the ST outbreak. All oil palm workers were given health education on ST and personal care while at the oil palm estate, such as wearing thick clothes, shoes, and insect repellent. Rat traps were installed in the area, and workers were advised to immediately seek treatment at a clinic/health facility if they had any symptoms.

The timeline of investigations was summarized in Table 1. It started with the Health District Office (HDO) receiving two notifications of leptospirosis between 7 and 11 December 2023. The onset of the index case (Patient 1M) was on 3rd December 2023, while the second case (Patient 2M) followed two days later. Case investigations at the oil palm estate were undertaken the next day, and a probable leptospirosis outbreak was declared. A couple of days later, three more cases (Patients 3M, 4F, and 5M) with a similar illness were reported with positive leptospirosis rapid test results. Following that, active case detection (ACD) was conducted on 20 December 2023 and noted that Patient 2M had a history of maculopapular rash. After a discussion with Pahang Epidemic Intelligence Program (EIP) specialist ST was added as one of the differential diagnoses besides leptospirosis, malaria, and melioidosis. On 27 December 2023, *Rickettsia* spp. and *Orientia* spp. blood and urine PCR tests were requested. Samples for the IIP test were also sent both for acute and convalescence (2 weeks apart) tests for ST. Positive results were seen in urine PCR for the index case and in serology (blood) tests in the other four cases. All cases were notified as ST.

## 6. Methods

### 6.1. Laboratory Investigation

#### 6.1.1. Molecular Detection by Real-Time Quantitative PCR (qPCR) and Nested PCR

The urine samples of the patients were sent to the Bacteriology Unit, Institute for Medical Research, which serves as the reference laboratory for Rickettsial diseases in Malaysia. Genomic DNA was extracted using the DNeasy^®^ Blood and Tissue Kit (Qiagen, Hilden, Germany) following the protocol as recommended by the manufacturer. The DNA was eluted in 50 µL AE elution buffer and stored at −40 °C until use. A qPCR assay targeting the 47 kDa periplasmic serine protease *htrA* gene was performed using the primers and probe as previously described [6,7,8]. Briefly, a total of 25 µL of reaction per tube was prepared, including 6.25 µL of master mix (4X CAPITALTM qPCR Probe Master Mix (Biotech Rabbit, Heidelberg, Germany) and 1 µL of each primer and probe. The two-step qPCR reaction was performed on the QuantStudio™ 6 Flex Real-Time PCR system (Thermo Fisher Scientific, Waltham, MA, USA) at initial denaturation: 94 °C for 5 min followed by 40 cycles of denaturation at 94 °C for 5 s and annealing at 60 °C for 30 s.

The positive DNA from qPCR was confirmed with nested PCR [8,9,10,11,12]. Two rounds of PCR were used to amplify the *tsa56* gene using PCRBIO Ultra Mix (PCR Biosystems, Phoenix, AZ, USA). First, the outer primer set was used for PCR amplification. The product from the first PCR was used as the template for the second PCR to amplify the final targeted fragment. The PCR conditions for both first and second PCR: initial denaturation at 94 °C for 2 s, denaturation at 94 °C for 30 s, annealing at 57 °C for one min, and extension at 72 °C for one min for 35 cycles and final extension at 72 °C for 10 min. The product was then visualized on a 2% agarose gel by the Bio-Rad ChemiDoc Touch Image System (Bio-Rad, Hercules, CA, USA).

#### 6.1.2. Genotyping

PCR-positive product was sent for sequencing (Apical Scientific, Shah Alam, Malaysia). The *tsa56* partial gene sequence (483 bp) of this study was compared with other known clinical OT genotypes available from GenBank (Supplementary Table S1). A phylogenetic tree was generated in MEGA 11 using the maximum likelihood method [13]. Bootstrap analysis with 1000 repetitions was performed to assess the robustness and reliability of the tree branching.

#### 6.1.3. Rickettsial Serology Testing by Indirect Immunoperoxidase Test

The IIP test for rickettsial serology was performed on the serum samples to detect the presence of immunoglobulin M (IgM) and immunoglobulin G (IgG) against rickettsial disease, i.e., ST, endemic typhus (ET), and tick typhus (TT), detecting *Rickettsial* spp. related to spotted fever group typhus (SFGR) [14]. Briefly, the sera were diluted at 1:50 to 1:1600 and incubated on slides coated with rickettsial antigens for 30 min at 37 °C. After washing with phosphate-buffered saline (PBS), the slides were incubated with peroxidase-conjugated IgM and IgG (Dako, Carpinteria, CA, USA) for 30 min at 37 °C. After washing with PBS, the slides were stained with 3,3-diaminobenzidine tetrahydrochloride (DAB) (Sigma, St. Louis, MO, USA) in the dark and briefly counterstained with methylene blue. The slides, upon washing and mounting, were observed under a light microscope at 40× (Olympus). The results were interpreted based on the presence of brown precipitates at dilutions as follows: <1:50 showed no evidence of active infection, and ≥1:100 and ≤1:400 gave results consistent with past or present infection. Confirmation of recent rickettsial infections by IIP is marked by ≥1:400 in a single sample or a four-fold increase in titers in a convalescent sample compared to the initial sample in 2–4 weeks [5,14].

### 6.2. Entomology Investigation

#### 6.2.1. Collection of On-Host Chiggers

An entomology investigation was carried out to examine the presence of rodents at the oil palm estate. A total of 28 mouse traps were placed at the outbreak site during two periods: from 15 to 20 December 2023 and from 20 to 25 February 2024. Trapping of small mammals using 28 wire traps was conducted in and around the houses of the index cases and several potential areas of infection for 5 consecutive nights. The captured rodents were euthanized, and special attention was given to the animals with chiggers attached to different body parts, including ears, abdomen, and legs. On-host chiggers were collected using an applicator stick from their animal hosts and stored in a Bijou bottle containing 70% ethanol and labelled individually according to a specific number assigned to each animal.

#### 6.2.2. Screening of *Orientia* spp. DNA

The chiggers were pooled (10–15 individuals/pool) according to the same small mammal hosts, and DNA was extracted using the DNeasy Blood and Tissue kit (Qiagen, Hilden, Germany) as recommended by the manufacturer. Nested PCR (nPCR) was performed to detect OT DNA in chiggers using species-specific primers targeting the 56 kDa type-specific antigen gene as described by Furuya et al. [10]. The PCR products were separated by electrophoresis at 90 V for 90 min in 1.5% agarose gels, stained with RedSafe DNA Stain (Thermo Fisher Scientific, Waltham, MA, USA), and visualized under a gel imaging system (UVP ChemoStudio Analytikjena, Jena, Germany).

### 6.3. Ethics Statement

This study was registered with the National Medical Research Register (NMRR) and ethically approved by the Medical Research and Ethics Committee (MREC), Ministry of Health, Malaysia, with reference number NMRR-24-02585-ZD9 on 2 October 2024. All samples included in this study were post-diagnostic specimens collected as part of routine clinical care and processed according to standard protocols. Prior to inclusion in the study, all samples were de-identified and anonymized to ensure patient confidentiality. As such, the requirement for informed consent was waived.

## 7. Results

### 7.1. Demography, Clinical Presentations, and Laboratory Results

Of the three hospitalized cases (patients 1M, 2M, and 3M), the index case (patient 1M) and patient 3M had raised creatinine kinase and transaminitis. Meanwhile, patients 2M and 3M had thrombocytopenia. All cases had negative dengue, malaria, and leptospirosis confirmatory test findings. The index case had a positive urine PCR test for ST. Table 2 showed the demography and clinical findings of in all five suspected cases, while Table 3 summarized details of the laboratory findings.. The details of IIP Rickettsial serology test results were described in Table 4. All cases showed a four-fold rise in paired serum for ST IgG titers. In fact, four cases showed very high titers beyond 1:1600 for ST compared to endemic typhus (ET) and tick typhus (TT). This finding confirmed STs recent infections and cross-reactivity in ET and TT result.

### 7.2. PCR and Genotyping of Orientia tsutsugamushi in Chiggers and Patients

A total of 28 wire traps were deployed in the potential areas of infection for four consecutive nights. Of that, five animals were captured (capture rate: 4.5%), and four were infested with chiggers, resulting in a total of 70 individual chiggers collected. Only four pools (15 individuals/pool) of chiggers were screened for the presence of OT using PCR analysis; however, all pools were negative.

**Figure 2:**
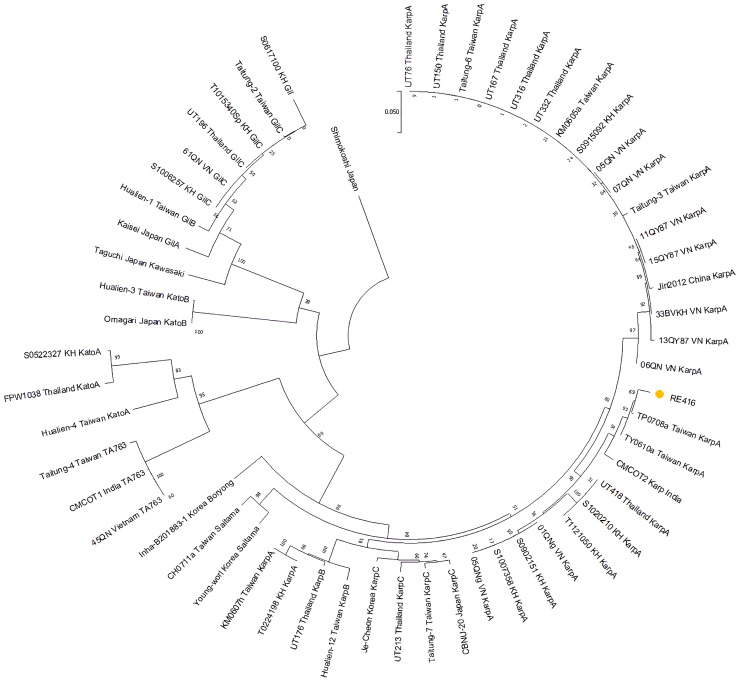
OT genotyping based on *tsa56* gene. The OT-positive sample from urine, RE416 (labeled in yellow), was aligned with other known OT genotypes, *n* = 56 (KarpA, KarpB, KarpC, Saitama, Boryong, TA763, Kato A, Kato B, GilA, GilB, GilC, Kawasaki, Shimokoshi), and a maximum-likelihood tree was generated. Bootstrap analysis with 1000 repetitions was performed to assess the robustness and reliability of the tree branching.

Phylogenetic analysis based on a total of 57 partial *tsa56* genes, including RE416 (in this study), known genotypes: Karp (n = 36), Gilliam (n = 8), Kato (n = 5), TA763 (n = 3), Saitama (n = 2), Boryong (n = 1), and Shimokoshi as the outlier (Supplementary Table S1). The tree was portrayed as a circular tree (Figure 2) and showed that sample RE416 clustered within the Karp population. RE416 was categorized as KarpA genotype with reference to grouping analysis by Kim et al. [15].

## 8. Discussion

Diagnosing ST remains a critical challenge in tropical countries, as the clinical presentations are similar to other tropical illnesses. Nevertheless, it should not be omitted from differential diagnoses, especially when tests for malaria and dengue fever yield negative results, even in the absence of an eschar. Furthermore, patients with positive IgM antibody rapid tests for leptospirosis should undergo confirmatory testing using the microscopic agglutination test (MAT), as the presence of IgM antibodies against *Leptospira* spp. may yield false-positive results in populations living in leptospirosis-endemic areas or due to cross-reactivity with other tropical diseases. Cases demonstrating poor response to beta-lactam antibiotics, including third-generation cephalosporins commonly used for leptospirosis treatment, should raise suspicion for rickettsial diseases. In such instances, the inclusion of doxycycline in the treatment regimen should be strongly considered.

Several important lessons emerge from this outbreak. Of particular interest was the case of the most severe patient, Patient 1M, who was diagnosed with ST when his urine PCR was positive and supported with a four-fold rise in antibody titers against the Karp, Kato, and Gilliam strains of OT. The urine sample was obtained 14 days after onset, which was previously used for Leptospira PCR, whereas blood samples tested for ST PCR were taken at 24 days after onset, which was not suitable for nucleic acid detection. There were two PCR assays being used for the diagnosis, i.e., nested PCR for the 56 kDa gene and a real-time qPCR targeting the 47 kDa gene, which both hold very high sensitivity and specificity for detecting OT DNA at a Cq value of 30, which was slightly lower than that of the positive control (Cq value of 32) [16,17].

The utilization of urine specimens for ST PCR testing is uncommon. Nevertheless, a study conducted in Mexico successfully identified *Rickettsia rickettsii* in suspected cases of Rocky Mountain Spotted Fever using urine PCR, with all cases subsequently confirmed through indirect immunofluorescence assay (IFA) [18]. Similarly, a study from Myanmar reported a single case, out of 671 patients presenting with acute febrile illness (AFI), that tested positive for the 47 kDa gene by real-time qPCR in urine. No cases were positive for Rickettsia TG or SFTG infection by urine rtPCR in this cohort [19]. Currently, no other published studies have documented positive ST PCR detection in human urine samples or in ST case with AKI, although ST is a recognizable aetiology with AKI complication [20]. In animal models, a limited number of studies have detected OT DNA within the renal tissue of rodents, as well as in organs including the spleen, liver, heart, and lungs, highlighting the potential involvement of the kidneys in disease pathology [21,22]. The study in Myanmar concluded that urine samples offered limited diagnostic utility for ST PCR, demonstrating markedly lower sensitivity compared to other sample types such as eschar, buffy coat, and whole blood. Eschar was reported to have the highest sensitivity, followed by buffy coat and whole blood. However, the prevalence of eschar differs according to geographical regions [17,18]. The sensitivity of ST PCR in urine was only 3.9%, compared to 30.8% in PCR from hemoculture fluid against IFA-confirmed cases, with similarly suboptimal specificity [18]. Several factors may contribute to the low sensitivity observed in urine-based ST’ PCR. The inherent dilutional nature of urine may reduce the already low concentrations of pathogen-derived nucleic acids. Furthermore, the presence of fragmented and degraded DNA, which may be filtered through the kidneys, particularly in patients with AKI, necessitates the optimization of extraction methods and the use of shorter amplicon targets to enhance detection. Additionally, PCR inhibitors such as urea and urinary crystals may compromise DNA stability during storage, especially if EDTA stabilization is not employed. Pre-admission antimicrobial use could further reduce pathogen load and negatively affect diagnostic sensitivity [18]. Although this report represents the first detection of OT DNA in urine from a patient with AKI, future research should include larger case-control studies with serial urine testing, particularly focusing on patients with renal complications such as vasculitis, tubular invasion, or renal failure. These studies could also investigate the optimal timing of specimen collection to capture intermittent shedding of OT DNA or explore alternative biomarkers for potential point-of-care testing. Ultimately, while urine PCR is unlikely to replace standard diagnostic specimens for ST, it could be integrated into a multi-sample molecular testing strategy to improve overall diagnostic sensitivity and yield.

The advancement in genome sequencing has led to divergence of the three main prototype genotypes (Karp, Kato, and Gilliam) to more variations [15]. An additional insight into the genotype level showed that the PCR-positive sample (RE416) of the KarpA genotype is clustered together with Taiwan’s (2006–2007) and India’s (2021) OT KarpA genotypes. It is surprising that our Malaysian isolate was more related to distant regions compared to neighboring countries within Southeast Asia. This could be due to the ease of traveling in the current era, resulting in possible importation of OT genotypes from other regions. Nevertheless, RE416 is still grouped with OT KarpA from Vietnam, Thailand, and Cambodia. OT Karp genotype is the most prevalent genotype in endemic regions compared to the Gilliam, Kato, TA763, and others [15,22]. It is still unsure of the pathogenicity and virulence of different OT genotypes. The degree of disease severity may be associated with virulence factors of different genotypes and DNA load [23]. Long et al. had reported that patients presenting OT Karp genotypes had significantly longer hospital stays and higher risk of developing multiple organ dysfunction than Kato and TA763 genotypes [24]. Wang et al. also reported that patients infected with the OT Karp genotype showed significantly higher DNA load compared to Gilliam and TA763 [25]. In vitro inoculation into mice experiments also provided evidence that Karp infection causes greater disease severity, mortality, pathologic lesions, and bacterial burdens in the lung and spleen [9,26]. Although there is limited information on the circulating OT genotypes in Malaysia, the Karp genotype is no stranger in Malaysia and has been reported [27,28]. A survey on the circulating clinical OT genotypes in Malaysia will aid in providing significant data on the association of disease severity and genotypes.

There are several reasons the entomology investigation was not successful. The main reason that may contribute to the time of the sampling is that it was performed shortly after a flood. This factor may have disrupted chigger habitats and reduced their numbers. Flooding can temporarily wash away chiggers or alter microhabitats and host availability, lowering both population density and the proportion of infected individuals. These disruptions may explain the failure to detect pathogen DNA despite confirmed human cases in the area [29]. Another important factor may be due to overemphasizing on-host chiggers only, excluding free-living ones from soil or vegetation. As the larval stage before host attachment, free-living chiggers may differ in infection rates and serve as key reservoirs of the pathogen. Relying solely on on-host sampling provides a limited view of the chigger population and may miss infected free-living individuals. This could lead to an underestimation of OT prevalence in chiggers and explain negative PCR results [30]. The needs of pooling chiggers for molecular detection may mix the infected and uninfected chiggers. The relatively low number of OT-infected chiggers in the pool would result in insufficient DNA concentration for positive detection in nested PCR. Future studies should include both on-host and free-living chiggers for a more comprehensive assessment of infection within the vector population. The low density of chiggers collected in the study areas likely contributed to the absence of detectable pathogen DNA, as some chiggers are not infected with OT [31]. In the current study, pooled chigger mites were also used. Therefore, any infection present at the individual mite may have been diluted by uninfected mites and could not be determined using conventional nested PCR. The use of more advanced molecular techniques such as quantitative PCR (qPCR) or digital PCR may improve detection of lower amounts of pathogen DNA and provide more accurate estimates of infection prevalence [32,33].

Although ST was discovered a century ago, it remains one of the most neglected tropical diseases in Malaysia, with limited awareness among healthcare professionals regarding its diverse clinical manifestations, diagnostic approaches, and optimal management strategies. This is particularly concerning given emerging evidence suggesting that the true prevalence of the disease may be higher than previously estimated [34,35]. A recent study in Teluk Intan identified ST as the cause of acute febrile illness cases in 24 out of 304 (8%), while ET and SFGR accounted for 7 (2%) and 11 (4%) cases, respectively [36]. The low awareness of ST among both the general population and healthcare workers contributes to delays in administering the appropriate antibiotics at initial patient encounters. Healthcare workers, particularly in suburban and rural areas, should be better informed about the disease and its associated symptoms. In India, the case fatality rate of ST has been reported at approximately 9%, with even higher mortality in cases involving secondary complications [37]. Early detection and diagnosis are critical for effective management, necessitating prompt identification of cases through meticulous evaluation of clinical signs and symptoms, complemented by laboratory diagnostic results [38]. In addition to awareness deficits, several other factors hindered effective management of the outbreak, including language barriers, environmental challenges, and landscape issues. Employing translators early in the diagnostic process could ensure accurate information gathering for better clinical outcomes. Environmental factors, such as monsoon flooding, exacerbated the spread of diseases by hindering hygiene practices and public health control activities. Preventing ST infection requires short-term vector control measures, including applying insecticides and managing vegetation to reduce vector populations. Personal protective strategies, such as wearing long-sleeved clothing, using insect repellents on skin and clothing, and avoiding direct contact with vegetation or bare ground, are critical in minimizing exposures of high-risk groups to chiggers, the primary vectors of the disease. These measures are crucial in endemic areas, especially during the monsoon seasons [39].

## 9. Conclusions

This cluster highlights the need to consider ST as a differential diagnosis in acute febrile illness alongside other tropical diseases. It also underscores the importance of continuous education for high-risk populations and healthcare workers to enhance case detection and reduce complications. Strengthening diagnostic capacity and integrating targeted surveillance efforts are crucial for timely identification of cases. Furthermore, future research should focus on understanding regional variations in its molecular epidemiology and evaluating the effectiveness of preventive strategies. These efforts collectively will contribute to reducing the public health burden of ST.

## Figures and Tables

**Table 1:** Timeline of investigations performed on a cluster of ST cases.

Year	12.2024											1.2025
Date	3rd	5th	7th	11th	12th	13th	15th	20th	22nd	26th	27th	9th
Onset of Index Case (1M)	X											
Onset of 2nd Case (2M)		X										
1st Notiification Leptospirosis Case			X									
2nd Notification of Leptospirosis Case				X								
ACD Leptospirosis					X							
Declaration of Probable Leptospirosis Outbreak					X							
Onset of 3rd Case (3M)					X							
Onset of 4th Case (4F)						X						
Entomology study was carried out							X					
PCR Urine for leptospirosis sent to IMR							X					
3rd Notification of Leptospirosis Case							X					
All result come negative include Urine PCR								X				
Discussion with EIP specialist									X			
Further history—positive history of maculopapular rash and resolved eschar (4F)										X		
PCR Rickettsia and IIP serology were taken											X	
Result IIP serology positive												X
Flood at the oil palm plantation									X	X	X	X

**Table 2:** Demography and clinical signs in positive cases.

Characteristic	*n* = 5 (100%)
Age group (years)	
20–34	4 (80.0)
35–60	1 (20.0)
Citizenship	
Citizens	3 (60.0)
Non-citizens	2 (40.0)
Gender	
Male	4 (80.0)
Female	1 (20.0)
Ethnic group	
Orang Asli	3 (60.0)
Foreigner	2 (40.0)
Status hospital admission	
Admitted	3 (60.0)
Not admitted	2 (40.0)
Clinical findings	
Fever	5 (100.0)
Myalgia	3 (60.0)
Headache	4 (80.0)
Cough	3 (60.0)
Loss of appetite	4 (80.0)
Shortness of breath	1 (20.0)
Eschar	1 (20.0)
Lymphadenopathy	1 (20.0)

**Table 3:** List of laboratory test results.

Laboratory Test		1M	2M	3M	4F	5M
*Biochemistry*						
Total white cell		17	13	7	Not done	Not done
Platelet		173	137	106	Not done	Not done
AST		120	61	547	Not done	Not done
ALP		84	47	595	Not done	Not done
Creatine kinase		5139	109	272	Not done	Not done
*Antigen and Antibody Detection*						
Dengue NS1, IgG, IgM Combo rapid test		Negative	Negative	Negative	Not done	Not done
Leptospirosis IgM serology test		Positive	Positive	Positive	Not done	Not done
Microagglutination test (MAT)		Negative	Negative	Negative	Not done	Not done
*Microscopy test*						
Blood film for malarial parasite (BFMP)		Negative	Negative	Negative	Not done	Not done
*Molecular Test*						
Leptospira PCR		Negative	Negative	Not done	Not done	Not done
ST PCR (Urine)	56 kDA gene	Positive	Negative	Not done	Not done	Not done
	47 kDA gene	Positive	Negative	Not done	Not done	Not done
ST PCR (Blood)	56 kDA gene	Negative	Negative	Not done	Not done	Not done
	47 kDA gene	Negative	Negative	Not done	Not done	Not done

**Table 4:** Rickettsial Serology Indirect Immunoperoxidase Test Results.

Antigen	Antibody	9 January 2024: 1st Sample					24 January 2024: 2nd Sample				
		1M	2M	3M	4F	5M	1M	2M	3M	4F	5M
S	IgG						‡	‡	‡	•	‡
T	IgM						‡	‡	‡		•
E	IgG						•	•			
T	IgM						•	•			
T	IgG							•	•		
T	IgM						•	•	•		•
	ST: scrub typhus			ET: endemic typhus				TT: tick typhus			
	<1;50			1;100			1;400			1;1600	
				1;200			1;800			>1;1600	

• Four-fold rise in IgG titer in paired sera which confirmed recent infection. ‡ Highest dilution beyond four-fold rise in dilution.

## Data Availability

The original contributions presented in this study are included in the article/Supplementary Materials. Further inquiries can be directed to the corresponding author.

## Supplementary Materials

The following supporting information can be downloaded at: https://www.mdpi.com/article/10.3390/tropicalmed10080208/s1, Supplementary Table S1. List of 57 tsa56 genes and their information used in this study.
